# Yes-Associated Protein 65 (YAP) Expands Neural Progenitors and Regulates *Pax3* Expression in the Neural Plate Border Zone

**DOI:** 10.1371/journal.pone.0020309

**Published:** 2011-06-08

**Authors:** Stephen T. Gee, Sharon L. Milgram, Kenneth L. Kramer, Frank L. Conlon, Sally A. Moody

**Affiliations:** 1 Department of Cell and Developmental Biology, University of North Carolina at Chapel Hill School of Medicine, Chapel Hill, North Carolina, United States of America; 2 National Heart, Lung and Blood Institute, National Institutes of Health, Bethesda, Maryland, United States of America; 3 Departments of Biology and Genetics, University of North Carolina at Chapel Hill, Chapel Hill, North Carolina, United States of America; 4 Department of Anatomy and Regenerative Biology, The George Washington University, Washington, D.C., United States of America; University of Colorado, Boulder, United States of America

## Abstract

Yes-associated protein 65 (YAP) contains multiple protein-protein interaction domains and functions as both a transcriptional co-activator and as a scaffolding protein. Mouse embryos lacking YAP did not survive past embryonic day 8.5 and showed signs of defective yolk sac vasculogenesis, chorioallantoic fusion, and anterior-posterior (A-P) axis elongation. Given that the YAP knockout mouse defects might be due in part to nutritional deficiencies, we sought to better characterize a role for YAP during early development using embryos that develop externally. YAP morpholino (MO)-mediated loss-of-function in both frog and fish resulted in incomplete epiboly at gastrulation and impaired axis formation, similar to the mouse phenotype. In frog, germ layer specific genes were expressed, but they were temporally delayed. YAP MO-mediated partial knockdown in frog allowed a shortened axis to form. YAP gain-of-function in *Xenopus* expanded the progenitor populations in the neural plate (*sox2^+^*) and neural plate border zone (*pax3^+^*), while inhibiting the expression of later markers of tissues derived from the neural plate border zone (neural crest, pre-placodal ectoderm, hatching gland), as well as epidermis and somitic muscle. YAP directly regulates *pax3* expression via association with TEAD1 (N-TEF) at a highly conserved, previously undescribed, TEAD-binding site within the 5′ regulatory region of *pax3*. Structure/function analyses revealed that the PDZ-binding motif of YAP contributes to the inhibition of epidermal and somitic muscle differentiation, but a complete, intact YAP protein is required for expansion of the neural plate and neural plate border zone progenitor pools. These results provide a thorough analysis of YAP mediated gene expression changes in loss- and gain-of-function experiments. Furthermore, this is the first report to use YAP structure-function analyzes to determine which portion of YAP is involved in specific gene expression changes and the first to show direct *in vivo* evidence of YAP's role in regulating *pax3* neural crest expression.

## Introduction

Yes-associated protein 65 (YAP) contains multiple protein-protein interaction domains and functions as both a transcriptional co-activator and as a scaffolding protein. YAP was first identified and named based on its association with the Src-family tyrosine kinase and proto-oncogene, c-Yes [1]. YAP is a founding member of the WW domain-containing protein family [2], [3]. The WW domain allows the binding of proteins containing a PPxY motif [4]. Proteins shown to bind to YAP via its two WW domains include: p53 family members (p73α, p73β, p63 [5]; Smad7 [6]; Runx2 [7]; and ErbB4 [8], [9].

**Figure 1:**
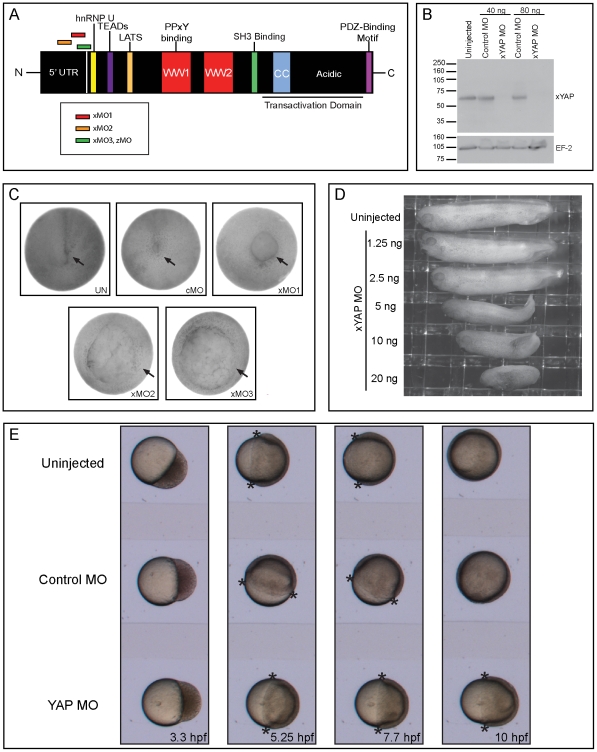
YAP morphant embryos exhibit defects in A-P axis elongation. (**A**) Frog and zebrafish YAP possess the ascribed functional and protein-protein interaction domains, including the TEAD-binding site (purple), the LATS phosphorylation site (orange), the two WW domains (red) that allow for PPxY binding, the Src Homology 3 (SH3)-binding domain (green), the coiled-coil region (blue), the transactivation domain (underline), and the PDZ-binding motif (pink). hnRNP U (yellow) binding has only been experimentally tested with human YAP, but related sites are in the fish and frog proteins. This diagram also illustrates the relative location of *Xenopus laevis* (x) and *Danio rerio* (z) MO-binding sites. (**B**) Injection of an equimolar cocktail of all three xYAP MOs at two concentrations (40 ng and 80 ng) resulted in efficient knockdown of endogenous, zygotic xYAP protein in stage 15 embryos as measured by western blot analysis. EF-2 expression from the same blot served as the loading control. (**C**) Three different xYAP MOs (80 ng; see A for binding sites) resulted in failed closure of the blastopore (arrows). (**D**) Reducing the concentration of the xYAP MO cocktail (left side) allowed blastopore closure, but resulted in dose-dependent A-P axis shortening. (**E**) Time-lapse video microscopy showed that zYAP MO (16 ng) injected embryos also exhibit perturbation in the completion of gastrulation. Asterisks mark the tissue front of epiboly movements. In uninjected and cMO-injected embryos, this front completely envelops the yolk by 10 hours post-fertilization (hpf). These fronts are still in the equatorial region in the 7.7–10 hpf YAP MO-injected embryos.

In addition to the two WW domains, YAP also contains other protein-protein interaction domains (Figure 1A). Proteins that interact at the N-terminus of YAP include hnRNP U, a nuclear ribonucleoprotein shown to be important for RNA polymerase II transcription [10], [11], the TEA domain-containing transcription factor (TEAD/TEF) family [12], and the Large tumor suppressor (LATS). The phosphorylation event involving LATS via the Hippo signaling pathway allows for the binding of 14-3-3, which leads to the subsequent sequestration of YAP to the cytoplasm [13]. At its C-terminus, YAP contains a postsynaptic density 95, discs large, and zonula occludens-1 (PDZ)-binding motif that allows for binding to PDZ domain-containing proteins.

Our initial interest in YAP came from the finding that YAP bound to the second PDZ domain of Na(+)/H(+) exchanger regulator factor 1/ezrin/radixin/moesin (ERM)-binding phosphoprotein of 50 kDa (NHERF1/EBP50) and co-localized to the apical membrane of polarized airway epithelia along with CFTR and c-Yes [14]. To determine the *in vivo* importance of this scaffolding complex, we used homologous recombination to remove YAP from the mouse and found that few embryos survived past embryonic day 8.5, a much earlier time point than would be expected for an associated lung development phenotype [15]. Detailed analyses of these mice illustrated that they suffered from defects in yolk sac vasculogenesis, chorioallantoic fusion, and anterior-posterior (A-P) axis elongation.

Given that YAP knockout mice struggled to progress normally through early development, in part because of nutritional deficiencies, we sought to better characterize a role for YAP during this time period by using embryos that develop externally: *Xenopus laevis* and *Danio rerio*. YAP morpholino (MO)-mediated complete loss-of-function prevented the completion of epiboly, delayed mesoderm induction, and severely impaired A-P axis elongation, phenotypes that were similar to YAP^−/−^ mice. YAP gain-of-function experiments in *Xenopus laevis* expanded the progenitors of the neural plate and neural plate border zone, while concomitantly inhibiting expression of later markers of tissues derived from the neural plate border zone (neural crest, pre-placodal ectoderm (PPE), hatching gland), as well as epidermis and somitic muscle. Through gain- and loss-of-function experiments and endogenous chromatin immunoprecipitations (ChIP) for YAP, we show that YAP directly regulates *pax3* expression via association with TEAD1 (N-TEF) and ultimately localizes to a highly conserved, previously undescribed, TEAD-binding site within the 5′ regulatory region of *pax3*. Finally, structure/function analyses revealed that the PDZ-binding motif of YAP contributes to the inhibition of epidermal and somitic muscle differentiation, but a complete, intact YAP protein is required for expansion of the neural plate and neural plate border zone progenitor pools.

## Materials and Methods

### Animal use and ethics statement

All experimental procedures described in this study followed the U.S. Public Health Service Policy of Humane Care and Use of Laboratory Animals and were approved by the Institutional Animal Care and Use Committee at the National Institutes of Health (NHLBI Animal Study Protocol: #H-0063), University of North Carolina at Chapel Hill (IACUC ID: 10-277.0), and the George Washington University (GWU Animal Study Protocol: #A-3205).

### xYAP and morpholinos

A *Xenopus laevis* full-length cDNA clone (XL211h05) of *yes-associated protein 65* (*xyap*) was obtained from the National Institute for Basic Biology (Japan) and sequenced in both directions (GenBank Accession #FJ979828). Three morpholinos, MO1 (GGA GGT GGG AGC TAG GAC AGC GG), MO2 (GGA GAG GAC GCG GTA GGA GAC TGT G), and MO3 (GGG CTC CAT GGC TGC GGG GAG GTG G), were designed to the 5′UTR of *xyap* for translational blocking (Fig. 1A; GeneTools). Two splice blocking and putative early translational truncation MOs, exon 1 (GTA GAG GAG CAT ATA CCT GCC GTG A) and exon 2 (CCT GCA AAG AAC AAG TGG GAC AAT A) (GeneTools) were designed across exon/intron boundaries. *In vitro* translation reactions were performed using the TnT Quick Coupled Transcription/Translation System (Promega), according to the manufacturer's protocol. Each MO (80 ng) was injected into *in vitro* fertilized 1-cell *Xenopus laevis* embryos according to established methods [16]. To observe phenotypes associated with lower MO concentrations (0, 1.25, 2.5, 5, 10, 20, and 40 ng total), a cocktail of all three (MO1, MO2, and MO3) translational blocking MOs was injected into *in vitro*-fertilized 1-cell sibling embryos.

### Cloning methods and constructs

For use in all of our gain-of-function analyses, we initially cloned an HA tag (ATG TAC CCA TAC GAT GTT CCA GAT TAC GCT) into the *Xho*I and *Eco*RV sites of the pSP64TXB vector so that proper expression could be detected by western blot analysis. *xyap* and *xtead1* were subcloned in frame with the HA tag into the *Eco*RV and *Not*I sites of the pSP64TXB-HA vector. A set of xYAP mutant constructs, which included a constitutively active form of xYAP (cActive xYAP) with a mutated LATS phosphorylation site (S98A), a deletion (aa 61–81) of the TEAD-binding site (xYAPΔTBS), a deletion (aa 78–161, aa 199–236) of the WW domains (xYAPΔWW), a deletion (aa 1–90) of the entire N-terminus (xYAPΔN), and a deletion (aa 455–459) of the PDZ-binding motif at the C-terminus (xYAPΔC), were also subcloned into the pSP64TXB-HA vector at the *Eco*RV and *Not*I sites.

### Gain-of-function analyses

For initial gain-of-function analyses, the animal poles of 1-cell *Xenopus laevis* embryos were injected with 2 ng of *in vitro*-transcribed *ha-xyap* mRNA (mMessage Machine, Ambion). For additional gain-of-function analyses, 2-cell *Xenopus laevis* embryos were co-injected with 1 ng of *in vitro*-transcribed *ha-xyap* or *ha-xyap* mutant mRNAs and 100 pg of *in vitro*-transcribed *nls-β-galactosidase* mRNA into the lateral, animal pole of one of the two blastomeres. Similarly, 100 pg of the *in vitro*-transcribed *ha-xtead1* (*xn-tef1*) [17] or 100 pg of *ha-xyap* mRNAs, were injected alone or in combination. Every mRNA injection was repeated 2–4 times per construct using different parental frogs to normalize against variation in genetic backgrounds and micropipette delivery. Results from these independent experiments were then pooled.

### Western blots

Embryos were injected at the 1-cell stage with a cocktail of the three (MO1, MO2, and MO3) translational blocking xYAP MOs (40 ng or 80 ng total) or the standard control MO (40 ng or 80 ng). These sibling embryos were allowed to develop until control embryos reached stage 15. Whole embryo lysates were snap frozen in a dry ice/ethanol bath and lysed in RIPA buffer (50 mM Tris, pH 8.0, 150 mM NaCl, 1% NP-40, 0.5% sodium deoxycholate, 0.1% SDS) containing a protease inhibitor cocktail (Roche). Subsequently, Freon (1,1,2-trichlorotrifluoroethane, Sigma-Aldrich) was used to remove the yolk from the samples, which were then boiled for 5 min and stored at −80°C. Total protein concentrations were quantified using the BCA Protein Assay Kit (Pierce), according to the manufacturer's protocol. Equal amounts of protein were loaded onto a 10% SDS-PAGE gel, separated by electrophoresis, transferred to a PVDF membrane, and probed for xYAP using an affinity purified rabbit anti-YAP antibody (1∶1000), which was generated against human YAP (274–454) [10]. The blots were then probed with a secondary HRP conjugated anti-rabbit IgG antibody (1∶10,000) (Jackson ImmunoResearch). The blots were incubated in stripping buffer (62.5 mM Tris, 2% SDS, 0.1 M β-mercaptoethanol, pH 6.7) and re-probed for elongation factor-2 (EF-2) using a goat anti-EF-2 antibody (1∶500) (Santa Cruz) and a secondary HRP-conjugated anti-goat IgG antibody (1∶4000) (Jackson ImmunoResearch).

### zYAP and embryo manipulations

A *Danio rerio* full-length cDNA IMAGE clone 7066008 of *yes-associated protein 65* (*zyap*) (NM_001115121) was obtained from Open Biosystems. A zYAP MO (5′ CTC TTC TTT CTA TCC AAC TGA AAC C 3′) was designed to the 5′ UTR of *zyap* (GeneTools). *In vitro* translation reactions were performed using the TnT Quick Coupled Transcription/Translation System (Promega), according to the manufacturer's protocol.

1-cell embryos were injected with 16 ng of the zYAP MO, a standard control MO (GeneTools), or 300 or 600 pg of *in vitro*-transcribed *ha-zyap* mRNA. Once embryos reached the 1000–2000-cell stage, their chorion membranes were removed and embryos were placed on a custom fitted imaging mold (kindly supplied by Dr. Sean Megason) [18] for time-lapse videography. Embryos were subsequently allowed to progress to the prim-11 stage and fixed.

### qPCR

*Xenopus laevis* embryos were injected with 80 ng of the translational blocking xYAP MO cocktail or a control MO at the 1-cell stage. When sibling control embryos reached stage 11, total RNA was isolated using the Trizol reagent (Invitrogen), according to the manufacturer's protocol. RNA was quantified using a RiboGreen RNA quantitation kit (Invitrogen), according to the manufacturer's instructions. Total RNA (2 µg) was reverse transcribed using Vilo cDNA synthesis (Invitrogen), according to the manufacturer's protocol. Then, qPCR was performed on a 7900HT 380-well block Real-Time PCR system (Applied Biosystems) using Maxima SYBR green qPCR master mix (Fermentas) on serial dilutions of the RT product to ensure the efficiency of amplification with each primer set was within 10% of one another. A relative quantification study was performed using 5 µL of a 1∶100 dilution of the RT product, which was amplified using gene-specific primers: *brachyury* (Forward: 5′ TCT CTT TCA CAT GCT GTG CC 3′, Reverse: 5′ GTG CCG TGA CAT CAT ACT GG 3′); *goosecoid* (Forward: 5′ CAC ACA AAG TCG CAG AGT CTC 3′, Reverse: 5′ GGA GAG CAG AAG TTG GGG CCA 3′); *wnt8* (Forward: 5′ TAT CTG GAA GTT GCA GCA TAC A 3′, Reverse: 5′ GCA GGC ACT CTC GTC CCT CTG T 3′); *nodal-related 3* (*nr3*) (Forward: 5′ CGA GTG CAA GAA GGT GGA CA 3′, Reverse: 5′ ATC TTC ATG GGG ACA CAG GA 3′); *siamois* (Forward: 5′ AAG ATA ACT GGC ATT CCT GAG C 3′, Reverse: 5′ GGT AGG GCT GTG TAT TTG AAG G 3′); *sox17α* (Forward: 5′ GCA AGA TGC TTG GCA AGT CG 3′, Reverse: 5′ GCT GAA GTT CTC TAG ACA CA 3′); *sox11* (Forward: 5′ GGC TCT GGA TGA GAG TGA CC 3′, Reverse: 5′ TGA TGA AGG GGA TTT TCT CG 3′); *h4* (Forward: 5′ GGG ATA ACA TTC AGG GTA TC 3′, Reverse: 5′ CAT GGC GGT AAC TGT CTT C 3′).

### In situ hybridization and β-galactosidase staining

*Xenopus laevis* embryos were fixed in MEMFA, stained for expression of a NLS-β-galactosidase lineage tracer, and processed for whole mount *in situ* hybridization according to standard protocols [16], [19]. Anti-sense DIG-labeled RNA probes were synthesized from the following plasmids: *chordin* (*Eco*RI, T7) [20], *eomesodermin* (*Xho*I, T7) [21], *brachyury* (*Cla*I, T7) [22], *vent2* (*Eco*RI, T7) [23], *not* (*Hind*III, T7) [24], *sox2* (*Hind*III, T7) [25], *neuroD* (*Xho*I, T3) [26], *n-tubulin* (*Bam*HI, T3) [27], *p27^Xic1^* (*Bam*HI, T7) [28], *sox11* (*Sal*I, T3) [29], *six1* (*Not*I, T7) [30], *notch* (*Cla*I, Sp6) [31], *hes1* (*Sal*I, T7) (Open Biosystems, BC070988), *zic1* (*Eco*RI, T3) [32], *foxD3* (*Bam*HI, T3) [33], and *pax3* (*Sal*I, T7) [25]. A full-length probe for *myoD* was PCR amplified from a *Xenopus laevis* (stage 19–26) cDNA library, which was kindly provided by Dr. Aaron Zorn, using the following primers: forward (GGA CTA GTA TGG AGC TGT TGC CCC CAC CAC TG) and reverse (CGG AAT TCC TAT AAG ACG TGA TAG ATG GTG CTG), and subcloned into the pBluescript SK(−) vector at the *Spe*I and *Eco*RI sites. This *myoD* probe was then synthesized as described above (*Spe*I, T7). Embryos derived from independent microinjection experiments were processed independently for ISH to normalize against processing variations.

### Chromatin Immunoprecipitation (ChIP)

ChIP assays were performed with the ChIP-IT Express kit (Active Motif) with some modifications. Three hundred stage 14–16 *Xenopus laevis* embryos were incubated, with gentle rolling, in 10 ml of 1% formaldehyde/0.1× modified Barth's solution (MBS) for 30 min at room temperature to crosslink genomic DNA and protein complexes. Crosslinking was stopped by incubating the embryos in 125 mM glycine/0.1× MBS with gentle rolling. Following two washes in 0.1× MBS, the embryos were snap-frozen and stored at −80°C. Chromatin was sheared with a Misonix 3000 cup horn by repeating 6 cycles of 30 sec: 1 sec pulse, 0.5 sec off at a power of 5. Samples rested on ice for 1 minute between each cycle. Shearing efficiency was determined by resolving a reverse-crosslinked, precipitated sample of chromatin on a 1% agarose gel. This sample was quantified using a Nanodrop, and 12.5 or 25 µg of chromatin was subsequently immunoprecipitated for 4 hours with 2 mg of affinity-purified YAP antibody or rabbit IgG (Genscript) in the presence of 0.25 mg/ml BSA and 0.1 mg/ml herring sperm DNA. Beads were washed once with ChIP buffer 1 (Active Motif) and twice with ChIP buffer 2 (Active Motif) prior to elution and proteinase K treatment. Five percent of the eluate was used to amplify the *pax3* promoter TEAD-binding site region using the following *xpax3*-specific primers: forward (GCC TGA CAA TGG CAC CTT AT) and reverse (AGG CGC ACT TGT GTG ATT C). For subcloning this region, a proofreading DNA polymerase (cloned *Pfu* DNA polymerase, Stratagene) was used to PCR amplify the product from the isolated YAP co-immunoprecipitated *Xenopus laevis* genomic DNA. This PCR product was then gel-purified from a 1% agarose gel. Alanines were then added back to the ends using a non-proofreading DNA polymerase (Jumpstart Taq polymerase, Sigma-Aldrich). The products were then ligated into the pCRII-TOPO vector (Invitrogen) and sequenced.

## Results

We previously showed that YAP^−/−^ mice were embryonic lethal and exhibited severe developmental abnormalities that included defects in yolk sac vasculogenesis, chorioallantoic fusion, and A-P axis elongation [15]. Given that these defects could be due to nutritional deficiencies, we sought to better characterize a role for YAP during early development by using *Xenopus laevis* and *Danio rerio*, animal models for which the nutritional needs of the embryos are self-contained. In addition, these embryos permit easy knockdown of targeted protein expression via injection of gene-specific MOs and efficient gain-of-function assessment via mRNA injections.

### YAP is required for progression through gastrulation

The full-length *Xenopus laevis yap* (*xyap*) EST encodes a protein that is 78% identical to mouse YAP and contains all the described protein-protein interaction domains, as well as the transcriptional activation domain (Figure 1A). Isolation of *Xenopus laevis* genomic DNA and subsequent PCR validated that our RT-PCR primer design amplified a PCR product across exon-intron boundaries (data not shown). RT-PCR and western blot analyses revealed that xYAP mRNA and protein are maternally expressed in the unfertilized egg through blastula stages, and are abundantly expressed from the onset of zygotic transcription (at mid-blastula transition) through tadpole stages (Figure S1). These results are consistent with reports of ubiquitous maternal mRNA expression in *Xenopus tropicalis*, zebrafish and mouse, and widespread zygotic expression in multiple neural, neural crest, and mesoderm derived tissues, but limited expression in endoderm derived tissues of the post-gastrulation embryo [15], [34], [35], [36], [37].

Three xYAP MOs were designed around the translational start site (Figure 1A) and their efficacies were confirmed *in vitro* (data not shown). An antibody directed against the C-terminus (274–454) of human YAP (hYAP) detected a band at the appropriate size from cold *in vitro*-translated xYAP product and stage 15 whole embryo lysates (data not shown). We used this hYAP antibody to test the efficacy of our xYAP MOs *in vivo*. Lysates from stage 15 MO-injected embryos showed efficient knockdown of endogenous, zygotic xYAP protein expression to undetectable levels (Figure 1B), thus effectively mimicking the genetic knockout achieved in mouse [15].

*In vitro*-fertilized sibling *Xenopus laevis* embryos that were injected with 80 ng of any one of these xYAP MOs at the one-cell stage failed to complete epiboly and close the blastopore (MO1, n = 200, 100%; MO2, n = 185, 100%; MO3, n = 191, 100%), while uninjected (n = 349) and control MO-injected (n = 231) embryos progressed through gastrulation unexpurgated (Figure 1C). Furthermore, these xYAP MO-injected embryos arrested at the open-blastopore stages, demonstrating incomplete epiboly. The same effect was observed using 40 ng (n = 725, 100%) and 80 ng (n = 352, 100%) of an equimolar cocktail of all three translation-blocking MOs (data not shown), both of which eliminated detection of endogenous, zygotic xYAP protein expression (Figure 1B). A similar, but less robust, phenotype was achieved with xYAP splice MOs targeted to exon 1 (n = 328, 76%) and exon 2 (n = 131, 77%). The less penetrant phenotype observed with the xYAP splice MOs correlated with their reduced efficiency at knocking down endogenous, zygotic protein expression (Figure S2). Together, these results demonstrate the specificity of the xYAP MOs.

Reducing the concentration of the xYAP MO cocktail below 40 ng resulted in partial endogenous, zygotic YAP protein expression (data not shown). This concomitantly allowed blastopore closure, but resulted in dose dependent A-P axis elongation defects (Figure 1D). Embryos injected with 1.25 ng (n = 113) or 2.5 ng (n = 142) of the xYAP translation blocking MO cocktail appeared unaffected, whereas embryos injected with 5–20 ng (5 ng, n = 152; 10 ng, n = 155; 20 ng, n = 163) of this cocktail did not progress through gastrulation as rapidly as their control siblings, and had progressively shortened body axes (Figure 1D). A similar phenotype was reported for a 5–7.5 ng YAP MO dose in zebrafish embryos [36].

Although the defective blastopore closure phenotype was reproducible using three different translational blocking xYAP MOs individually or in combination as well as two different splice blocking xYAP MOs, the phenotype was not rescued by co-injecting 2 ng of frog (*xyap*), mouse (*myap*), or human (*hyap*) mRNAs, even though they all were properly translated in *Xenopus laevis* embryos (Figure S3). Therefore, we tested whether knockdown of YAP in another animal model, using similar methods, would produce a similar phenotype. Jiang *et al*. previously showed that a low dose of YAP MO that reduced fluorescence of a YAP 5′UTR-eGFP reporter resulted in shortened embryos with small heads; however, these embryos were only analyzed at late stages (30–50 hpf) and thereby did not address the gastrulation defects observed in frog YAP morphants [36]. Therefore, we injected 16 ng of a zYAP MO into fertilized one-cell zebrafish embryos, which completely eliminates expression of zYAP in an *in vitro* translation assay (data not shown). Time-lapse videography showed that in the absence of zYAP epiboly movements were arrested (Figure 1E). While the tissue front of epiboly gradually closed around the yolk cell between 5.25–10 hpf in uninjected and control MO-injected embryos (n = 15 per each group), this closure was not achieved in YAP morphants (n = 15; 100%). Thus, in three different vertebrates, loss of early YAP function interferes with the developmental networks that regulate completion of gastrulation movements.

**Figure 2:**
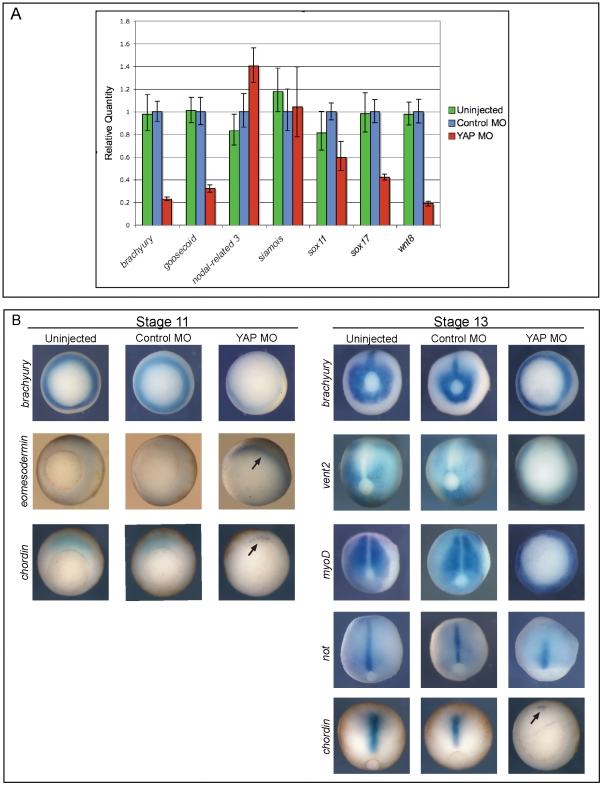
Germ layer markers are expressed in YAP morphant *Xenopus* embryos, but are temporally delayed. (**A**) qPCR analysis of mRNA from uninjected, control MO-injected, and xYAP MO-injected *Xenopus* embryos collected when controls reached stage 10.5/11. *brachyury*, *goosecoid*, *wnt8*, *sox11*, and *sox17* mRNA levels were reduced, *nodal-related 3* (*nr3*) mRNA levels were increased and *siamois* mRNA levels remained unchanged in xYAP morphant embryos. (**B**) *In situ* characterization of mesoderm gene expression in uninjected, control MO-, and xYAP MO-injected *Xenopus* embryos. xYAP morphant embryos express each gene in the correct location, but the spatial pattern resembles an earlier developmental stage. For example, *brachyury* expression in the stage 11 YAP MO embryos is only faintly detected and *brachyury* expression in the stage 13 YAP MO embryo is indistinguishable from the control stage 11 pattern. *chordin* expression in the stage 13 YAP MO embryo remains confined to the dorsal blastopore lip (arrow), as is normal at stage 11; it has not elongated with the axial mesoderm as is normal at stage 13. *eomesodermin* expression in the stage 11 YAP MO embryo remains on the surface in the uninvoluted mesoderm (arrow), whereas in controls, *eomesodermin*-expressing cells have migrated internally [21]. In the stage 11 panel, all views are vegetal; in the stage 13 panel, the views of *brachyury* and *vent2* embryos and of the YAP MO *chordin* embryos are vegetal and the remainder are dorsal.

To determine whether the MO-mediated gastrulation defects correlated with an effect on genes required for germ-layer formation, we performed qPCR analyses on well-established markers of each germ layer. Control MO (80 ng) or the xYAP MO cocktail (80 ng) were injected into one-cell *Xenopus laevis* embryos, and the embryos were collected when uninjected siblings reached mid-gastrulation (stage 11), a stage when germ layer markers are abundantly expressed. Genes normally expressed in the organizer at the onset of gastrulation were either unaffected (*siamois*) or moderately increased (*nodal-related 3*). In contrast, the expression levels of endodermal (*sox17*, p<0.013), neural ectodermal (*sox11*, p<0.021), and three out of five mesodermal (*brachyury*, p<0.013; *goosecoid*, p<0.011; *wnt8* p<0.018) genes were significantly reduced in YAP MO-injected embryos (Figure 2A). However, analyses of several mesodermal markers by *in situ* hybridization in *Xenopus laevis* showed that these quantitative changes resulted from delayed expression rather than loss of mesoderm induction. While *brachyury*, *eomesodermin*, and *chordin* expression was markedly reduced in xYAP MO-injected embryos compared to sibling stage 11 embryos (Figure 2B), at sibling stage 13 these genes, as well as several others, were expressed in patterns similar to control stage 11 embryos (Figure 2B, n = 14–25 per sample, 100% for all markers). Thus, eliminating endogenous, zygotic xYAP protein expression does not prevent mesodermal gene induction, but does delay the expression of a number of mesodermal genes. These results indicate that the lack of progression through gastrulation in YAP morphant embryos is not due to a failure in germ layer inductions.

### YAP gain-of-function also causes axis elongation defects

**Figure 3:**
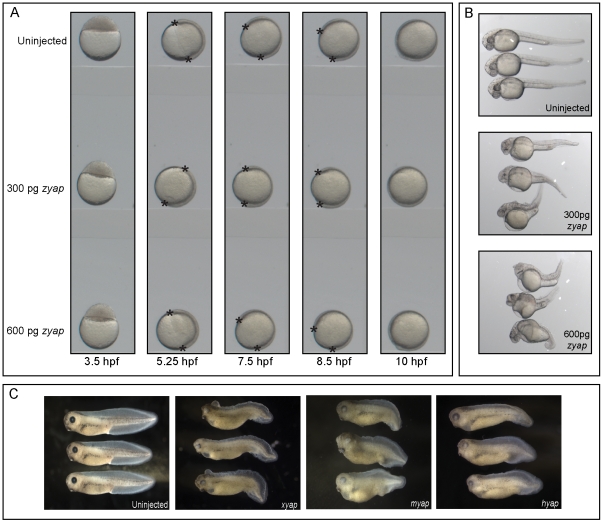
zYAP and xYAP gain-of-function results in similar body axis defects. (**A**) Time-lapse videomicroscopy shows that zYAP gain-of-function does not alter the timing of gastrulation movements, as evidenced by the progression of epiboly (asterisks mark the fronts of tissue movement around the yolk). (**B**) zYAP gain-of-function in *Danio rerio* embryos resulted in head and eye deformities and shortened, malformed body axes. Examples of two different mRNA doses are shown. (**C**) Injection of *Xenopus* (x), mouse (m), or human (h) *yap* mRNAs into *Xenopus* embryos all showed phenotypes similar to those in zebrafish.

From these results, we predicted that increasing YAP protein above endogenous levels may cause gastrulation to be completed more rapidly. However, time-lapse video recordings of gastrulation movements in zebrafish embryos injected with two different doses of *zyap* mRNA did not detect any differences in the amount of time required for epiboly movements to close around the yolk plug (Figure 3A, n = 5 per group). When these *zyap* mRNA injected embryos developed to later stages, however, significant perturbations were observed (Figure 3B, 300 pg, n = 111, 100%; 600 pg, n = 94; 100%), including shortened A-P body axis and perturbed somitic and head morphologies. Likewise, injection of *xyap*, *myap*, or *hyap* (2 ng) mRNAs all caused similar phenotypes in *Xenopus laevis* embryos (Figure 3C; n = 155 (*xyap*), n = 102 (*myap*), n = 93 *(hyap*), 100% affected). While these gain-of-function phenotypes resemble the reduced YAP phenotypes reported in zebrafish [36] and frog (Figure 1D), analysis of gene expression (next section) indicates that YAP gain-of-function and loss-of-function results in strikingly different effects. Since these late phenotypes likely result from early alterations in tissue patterning and/or A-P axis formation, and given that YAP is a well-described transcriptional co-activator, we predicted that altering YAP levels would have distinct effects on the expression of genes involved in these early developmental processes.

### YAP expands neural progenitors and inhibits neural differentiation

**Figure 4:**
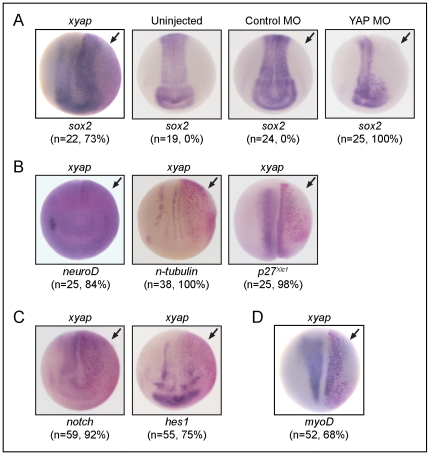
xYAP gain-of-function expands neural progenitor fields, while neural differentiation is inhibited. (**A**) The neural plate progenitor field marked by *sox2* expression (blue stain) was darker, longer, and/or wider on the xYAP-injected side (arrow, red β-gal staining) compared to the uninjected side of the same embryo. xYAP MO-mediated knockdown (40 ng) eliminated *sox2* expression on the injected side, whereas a control MO (cMO) did not. In this and all subsequent panels: n = sample size; % = frequency of the phenotype; arrow indicates injected side. (**B**) Three genes indicative of neural differentiation (*neuroD*, *n-tubulin*, *p27^Xic1^*) were inhibited by xYAP gain-of-function. (**C**) xYAP gain-of-function reduced *notch* and *hes1* expression. (**D**) Muscle differentiation marker, *myoD*, was reduced by xYAP gain-of-function. All views are dorsal-anterior.

Vertebrate A-P axis elongation is accomplished in part by elongation of the neural plate [38], [39], and in *Xenopus* and zebrafish neurulae, *yap* mRNA is robustly expressed in the floor plate and lateral borders of the neural plate [36], [40]. Therefore, we investigated whether neural progenitor fields were altered in YAP gain-of-function *Xenopus laevis* embryos. Given that our previous YAP gain-of-function experiments were injected into 1-cell *Xenopus laevis* embryos, we chose to more closely monitor where our injected mRNA ended up in the embryo by co-injecting *xyap* and *β-galatosidase* (β-gal; as a lineage tracer) mRNAs into one blastomere of the 2-cell embryo. The neural progenitor field, indicated by *sox2* expression, was expanded as evidenced by a darker, longer, and/or wider expression domain compared to the uninjected side of the same embryo (Figure 4A). Injection of the YAP MO cocktail (40 ng) caused a loss of *sox2* expression on the injected side (Figure 4A), indicating that the YAP gain-of-function and loss-of-function phenotypes are manifested early in body axis formation by different mechanisms. Expansion and perdurance of *sox2* expressed resulted in concomitant repression of neural differentiation markers (Figure 4B). *neuroD*, a bHLH neural differentiation transcription factor, *p27^Xic1^*, a cdk inhibitor shown to be important for cell cycle exit and subsequent neural differentiation [28], and *n-tubulin*, a post-mitotic neuron-specific tubulin, each were strongly repressed (Figure 4B). These results are consistent with a study in the chick neural tube demonstrating that phosphorylated YAP is expressed in the *sox2^+^* ventricular zone, and that electroporation of YAP increased the *sox2* ^+^ domain, decreased the number of neuron-specific tubulin positive cells and reduced cell cycle exit [41]. Interestingly, we also observed that the expression of a muscle-specific bHLH differentiation marker, *myoD*, was strongly repressed (n = 52, 68%) (Figure 4D) indicating that the effects of YAP were not confined to the neural ectoderm.

It is well documented that increased Notch expression and/or signaling correlates with increased numbers of neural progenitors, decreased differentiated neurons [31], [42], [43], and increased YAP expression [44]. Therefore, we were surprised to observe that xYAP gain-of-function reduced the mRNA levels of *notch* and *hes1*, a direct Notch signaling target gene [45], [46], [47] (Figure 4C). These results indicate that YAP's ability to repress neural differentiation is likely independent of Notch signaling.

**Figure 5 pone-0020309-g005:**
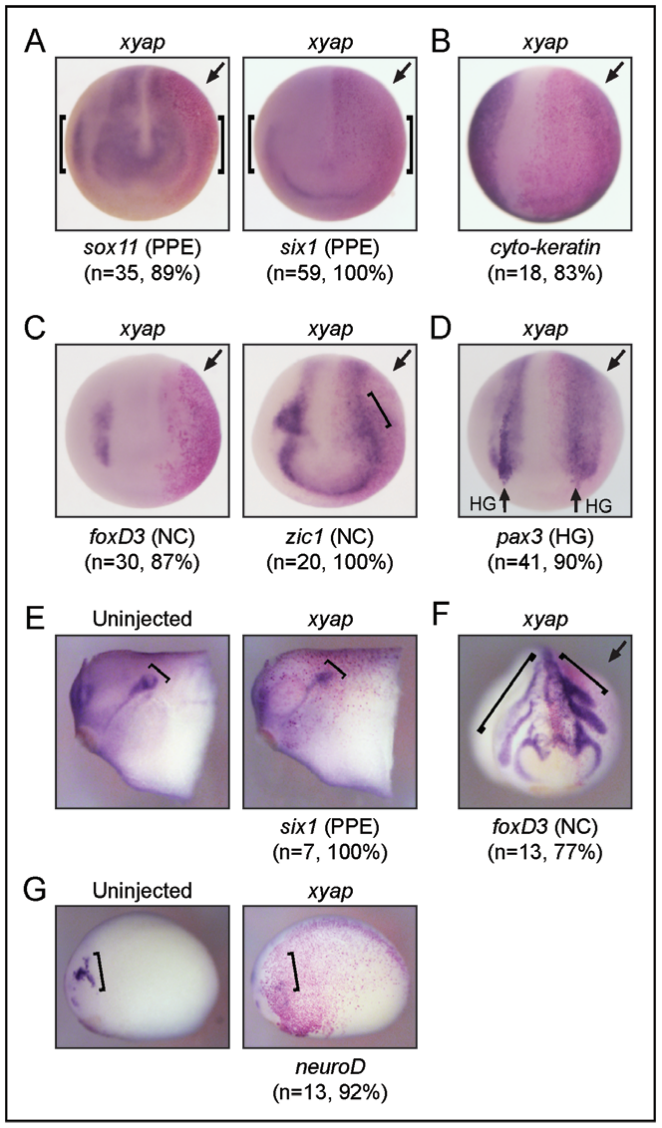
xYAP gain-of-function inhibits the expression of genes in the pre-placodal ectoderm, epidermis, premigratory neural crest, and hatching gland. (**A**) Genes expressed in the pre-placodal ectoderm (PPE), *sox11* and *six1*, are dramatically reduced on the xYAP-injected sides (arrows). Brackets indicate the laterally located PPE expression domains on both sides the embryos. Anterior views. (**B**) Expression of the epidermis-specific *cyto-keratin* gene is lost on the xYAP-injected side. Anterior view. (**C**) The expression of genes characteristic of premigratory neural crest (*foxD3*, *zic1* at bracket) are repressed on the xYAP-injected sides. Anterior-dorsal views. (**D**) *pax3* expression in the surface ectodermal A-P stripe, which indicates the hatching gland progenitors (vertical arrows, HG) is repressed on the xYAP-injected side. In contrast, *pax3* expression in the underlying neural crest progenitors is expanded (see Figure 6A). Dorsal view. (**E**) *six1* expression in the otocyst (bracket) is reduced on the xYAP-injected side (right panel) compared to the uninjected side (Un) of the same embryo (left panel). Side views, stage 26. (**F**) The migratory path of *foxD3*-expressing neural crest cells (indicated by brackets) is truncated on the xYAP-injected side (right). Frontal view, stage 24. (**G**) *neuroD* expression in the trigeminal placode (bracket) is reduced on the xYAP-injected side (right panel) compared to the uninjected side (Un) of the same embryo (left panel). Side views, stage 22.

**Figure 6:**
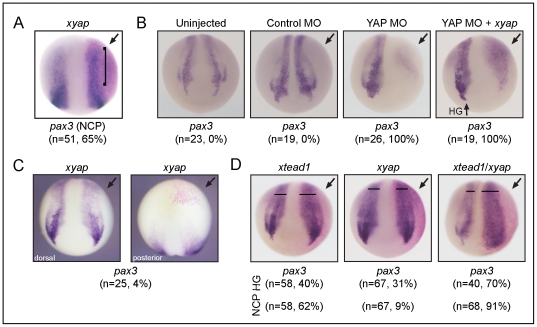
xYAP expands *pax3*-expressing neural crest progenitors. (**A**) The *pax3*-expressing neural crest progenitor field (NCP) is darker, longer, and/or wider (bracket) on the *xyap*-injected side. Dorsal view, stage 15. (**B**) xYAP MO-mediated knockdown (40 ng) eliminated *pax3* expression in both neural crest progenitors and hatching gland (HG) precursors. Addition of exogenous *xyap* (YAP MO + *xyap*) rescued *pax3* expression in neural crest progenitors (NCP), but not in hatching gland. Dorsal views, stage 17. (**C**) *xyap* mRNA injection into the precursor blastomere of the intermediate mesoderm does not cause *pax3* expansion on the injected side (left panel, dorsal view). Right panel (posterior view, dorsal is down) shows part of the lineage labeled clone (red) denoting the injected side. (**D**) *tead1* mRNA injection (100 pg) expands *pax3*-expressing neural crest progenitors (NCP) at a moderate frequency. *xyap* mRNA injection (100 pg) rarely expands this population. In combination (*tead1*/*xyap*, 100 pg each), this population is expanded in nearly every embryo. The repression of the *pax3*-expressing hatching gland progenitors (HG) also was greatest when TEAD/xYAP were co-expressed. Dorsal anterior views at stage 16.

The expansion of neural progenitors by increased YAP levels also reduced the expression domain of the differentiated epidermis, as marked by an epidermal-specific *cyto-keratin* [48] (Figure 5B). Because interactions between the neural plate and the epidermis lead to the formation of a neural plate border zone that gives rise to the precursors of the peripheral nervous system, the neural crest and the pre-placodal ectoderm (PPE) [49], we analyzed whether these tissues were properly induced at neural plate stages. While the *pax3* ^+^ domain surrounding the neural plate, which is required to specify neural crest [50], [51]), was expanded (Figures 5D, 6A), two genes expressed by premigratory neural crest (*foxD3*; *zic1*; [33], [51]) and two PPE genes (*six1*, *sox11*; [52]) were dramatically reduced (Figure 5A, B). In addition, the *pax3^+^* precursors of the hatching gland [51] were virtually eliminated (Figure 5D). Thus, in *xyap*-mRNA injected embryos the three different precursor populations that contribute to the formation of peripheral cranial structures did not develop at neural plate stages. To determine whether the placode and neural crest populations eventually develop, we targeted *xyap*-mRNA injections to the neural plate border zone blastomere precursors (blastomeres D12 and V12; [53]). At tail bud stages: 1) placode expression of *six1* was significantly reduced in the otocyst (Figure 5E) and the olfactory placode (data not shown); 2) *foxD3*-expressing neural crest cells were more dorsally located than in controls, indicating delayed migration into the periphery (Figure 5F); and 3) trigeminal placode expression of *neuroD* was reduced (Figure 5G). The perturbations in the differentiation of these precursors of peripheral cranial structures likely account for the cranial defects seen in the YAP gain-of-function late stage frog and fish embryos (Figure 3B, C).

### YAP cooperates with TEAD to expand pax3^+^ neural crest progenitors

While the *pax3^+^* hatching gland cells were virtually eliminated, *pax3* expression in the neural plate border zone that is required for neural crest specification [50] was extended, broadened, and/or stronger compared to the uninjected, control side of the embryo (Figures 5D, 6A). This expansion of the *pax3^+^* neural crest progenitor field was concomitant with a decrease in genes expressed by specified, premigratory neural crest (*zic1*, *foxD3*; Figure 5B), suggesting that increased YAP holds these cells in a progenitor, undifferentiated state longer than in an unmanipulated embryo. Consistent with these gain-of-function results, embryos that were injected with the xYAP MO cocktail (40 ng) exhibited a complete loss of *pax3* expression (Figure 6B). The loss of *pax3* in the neural crest progenitors, but not in the hatching gland precursors, could be rescued with *xyap* mRNA (Figure 6B).

In both frog and chick, the underlying mesoderm plays an important role in specifying the tissues derived from the neural plate border zone (reviewed in [54]. Therefore, it is possible that the expansion of the *pax3* ^+^ neural crest is secondary to YAP effects in the mesoderm. A recent study showed that it is the intermediate mesoderm, which lies directly underneath the neural plate border zone during neurulation, that is required for induction and maintenance of neural crest fate [55]. Therefore, we tested whether YAP activity in the intermediate mesoderm causes expansion of *pax3* neural crest expression by targeting *xyap*-mRNA injections to the blastomere lineage that gives rise to the intermediate mesoderm but does not contribute to the neural crest (blastomere V21, [53]). In these embryos, *pax3* ^+^ neural crest expression was expanded in only 1/25 embryos (Figure 6C), supporting the suggestion that YAP directly affects *pax3* expression.

Much of the *in vivo* transcriptional co-activator activity of YAP results from interactions with members of the TEAD transcription factor family [56]. Recently, Naye *et al*. characterized two *Xenopus* TEADs, *xtead1* (*xn-tef*) and *xtead3* (*xd-tef*) [17]. Injection of *xtead1* (100 pg) mRNA alone expanded the *pax3^+^* neural crest progenitors, while a low dose of *xyap* (100 pg) mRNA alone had little effect (Figure 6D). However, upon co-injection of equal amounts of *xtead1* (100 pg) and *xyap* (100 pg) mRNAs, the percentage of embryos with an expansion of *pax3^+^* neural crest progenitors was greatly increased (Figure 6D), indicating cooperativity between these proteins. Although TEAD gain-of-function alone expanded the *pax3^+^* neural crest progenitors, the above experiments show that YAP enhances this effect, and the YAP MO experiments indicate that YAP is required for this effect. Therefore, we predicted that YAP acts as a transcriptional co-factor with xTEAD1 in regulating *pax3* expression.

**Figure 7:**
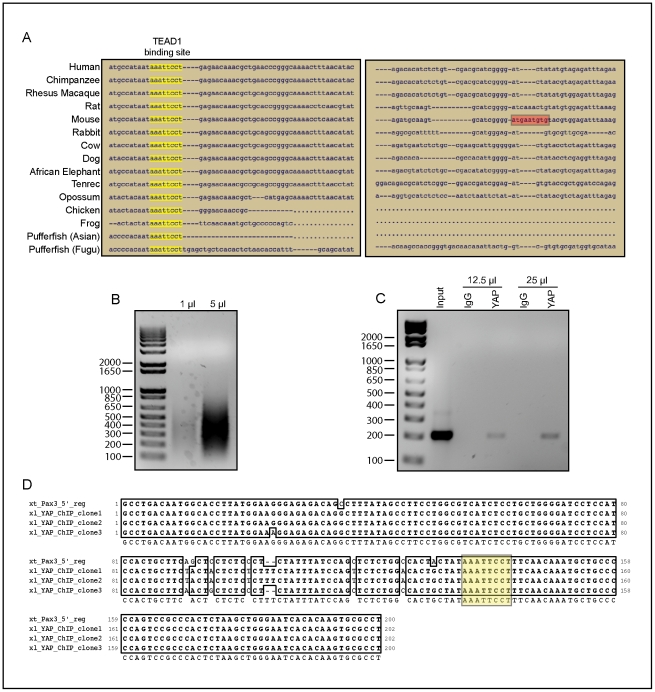
Endogenous xYAP resides at a novel 5′ regulatory region of *pax3*. (**A**) A highly conserved putative TEAD-binding site (yellow boxes) is present in the 5′ regulatory region of the *pax3* gene in 15 different vertebrates. A previously described mouse TEAD-binding site (red box) appears less conserved. (**B**) Chromatin isolated from 300 wild type stage 14–16 *Xenopus laevis* embryos was sheared to a size range of 150 to 900 base pairs. (**C**) Chromatin immunoprecipitations (ChIPs) from 12.5 µg or 25 µg of sheared chromatin immunoprecipitated a band at the expected size for the putative novel TEAD1-binding site region with the hYAP antibody but not with a control IgG antibody. (**D**) Sequencing of this band from three different clones verified that the genomic region pulled down by the hYAP anitbody contained the novel TEAD-binding site (yellow) when compared to the *Xenopus tropicalis* genomic sequence.

Results from a series of *pax3* promoter transgenic deletions led Milewski *et al*. to suggest that a TEAD-binding site within a neural crest enhancer region was responsible for neural crest expression of *pax3* [57]. However, we failed to find conservation of this previously described TEAD-binding site in the *Xenopus tropicalis* genome (Figure 7A). Using the genomic alignment and conserved transcription factor binding site prediction program, ConTra [58], a predicted TEAD-binding site that was highly conserved in 15 different vertebrates was identified 58 base pairs upstream of the previously described mouse neural crest enhancer TEAD2-binding site (Figure 7A). To demonstrate direct involvement of xYAP in the control of *pax3* transcription, we performed a ChIP analysis of the *xpax3* promoter from wild-type stage 14–16 *Xenopus laevis* embryo DNA that was sheared to an appropriate size (Figure 7B). Using primers made specifically to amplify the genomic region containing the conserved TEAD-binding site (yellow box in Figure 7A), endogenous xYAP co-immunoprecipitated with this region, illustrating the direct involvement of xYAP in regulating *xpax3* transcription (Figure 7C). This TEAD-binding site was specific since primers to another portion of the *pax3* promoter were not pulled down with the YAP antibody (Figure S4A). Likewise, a region of the *sox2* promoter, which possesses a putative TEAD-binding site, also failed to be pulled down with the YAP antibody (Figure S4B). To confirm the presence of the TEAD-binding site within the YAP chromatin-immunoprecipitated piece of *Xenopus laevis* genomic DNA, a proofreading *Taq* polymerase was used to amplify and subclone the product (Figure 7D). Interestingly, the conserved TEAD-binding site, but not the proposed mouse TEAD2-binding site, was located in this amplified fragment (Figure 7D).

### PDZ-binding motif of xYAP plays a role in epidermal and muscle differentiation

**Figure 8:**
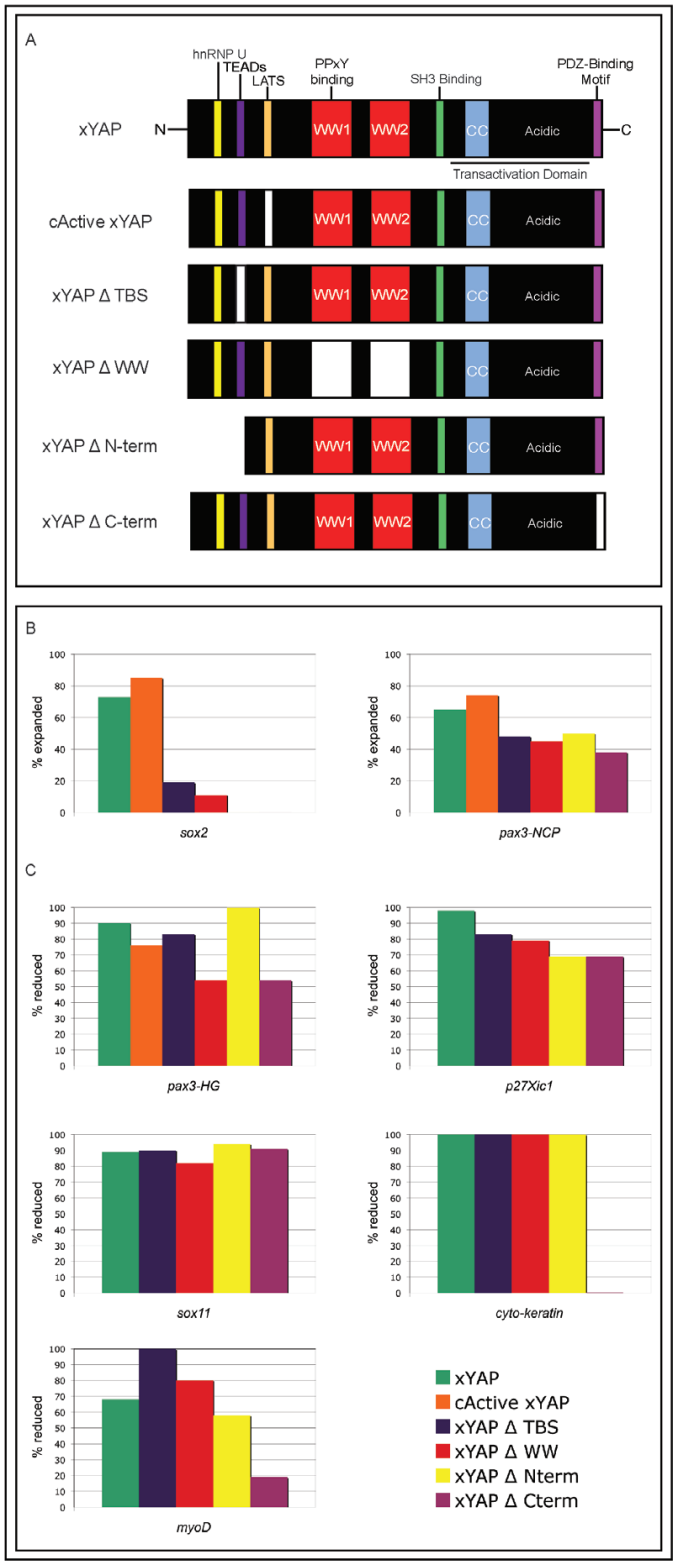
xYAP deletion mutants exhibit differential activities. (**A**) Cartoons of the xYAP mutants created to determine which protein-protein interaction domain(s) is important for the *in vivo* gain-of-function phenotypes described in Figures 4–6. Deletions or mutations are indicated by color loss: the TEAD-binding site (xYAPΔTBS, purple), the LATS phosphorylation site (cActive xYAP, orange), the two WW domains (xYAPΔWW, red), the N-terminus (xYAP, ΔN-term) containing both the hnRNP U and TEAD-binding sites, and the PDZ-binding motif (xYAPΔC-term, fuchsia) at the C-terminus. (**B**) The percentage of embryos showing expansion of *sox2*-expressing neural plate cells or expansion of *pax3*-expressing neural crest progenitor (NCP) cells after injection of each of the mutant forms of xYAP. Note that cActive xYAP, which prevents YAP from leaving the nucleus, is as effective as wild type YAP. However, all other mutant forms reduce this phenotype. Sample sizes are presented in Table 1. (**C**) The percentage of embryos showing reduced gene expression after injection of each mutant form of xYAP. Deletion of the WW domains or of the PDZ-binding motif interfered the most with repression of *pax3* ^+^ hatching gland (HG) progenitors. Loss of neural plate differentiation (*p27^xic1^*) and a PPE marker (*sox11*) were maintained at high frequencies with each xYAP mutant, indicating that interactions at one or more of the remaining domains are sufficient to downregulate these genes. However, xYAP-mediated loss of somitic muscle (*myoD*) and epidermal (*cyto-keratin*) differentiation was specifically reduced by deletion of its PDZ-binding motif.

To better define which protein-protein interaction domain of xYAP is responsible for the expansion of the neural plate and neural crest progenitors as well as the correlative inhibition of neural, hatching gland, PPE, epidermal, and somitic muscle differentiation, we performed a series of structure-function analyses whereby mutant forms of xYAP (Figure 8A) were expressed on one side of the embryo (Table 1). The constitutively active form of xYAP (cActive xYAP), in which the LATS phosphorylation site is mutated rendering the protein unable to exit the nucleus, caused expansion of neural plate (*sox2*) and neural crest (*pax3*) progenitors and reduction of *pax3* ^+^ hatching gland precursors at frequencies comparable to wild type xYAP (Figure 8B, C). These results confirm that the nuclear activity of YAP is required for these phenotypes. In contrast, deletion of the TEAD-binding site (TBS), WW domains, N-terminus, or C-terminus each resulted in a dramatic (*sox2*) to moderate (*pax3*-NCP, *pax3*-HG) reduction in the frequency of the respective phenotypes, indicating that an intact protein is required. These results implicate the importance of multiple binding partners, not solely the interaction with TEAD. In contrast, loss of neural plate differentiation (*p27^xic1^*) and formation of the PPE (*sox11*) were maintained at high frequencies with each xYAP mutant, indicating that interactions at one or more of the remaining domains are sufficient to downregulate these genes. Interestingly xYAP-mediated loss of epidermal (*cyto-keratin*) and somitic muscle (*myoD*) differentiation were specifically reduced by deletion of its PDZ-binding motif. These results implicate the involvement of a PDZ-containing interacting protein in the effects on these two tissues. The requirements for different YAP domains for the effects on these diverse embryonic tissues indicate that different binding partners are likely to mediate them.

**Table 1:** xYAP deletion mutants exhibit differential activities.

Probe	RNA Injected	% Change
*sox2*	xYAP	n = 22, 73% expanded
*sox2*	cActive xYAP	n-20, 85% expanded
*sox2*	xYAP Δ TBS	n = 42, 19% expanded
*sox2*	xYAP Δ WW	n = 62, 11% expanded
*sox2*	xYAP Δ N-term	n = 18, 0% expanded
*pax3* (NCP)	xYAP	n = 51, 65% expanded
*pax3* (NCP)	cActive xYAP	n = 19, 74% expanded
*pax3* (NCP)	xYAP Δ TBS	n = 68, 48% expanded
*pax3* (NCP)	xYAP Δ WW	n = 64, 45% expanded
*pax3* (NCP)	xYAP Δ N-term	n = 16, 50% expanded
*pax3* (NCP)	xYAP Δ C-term	n = 18, 38% expanded
*pax3* (HG)	xYAP	n = 41, 90% reduced
*pax3* (HG)	cActive xYAP	n = 17, 76% reduced
*pax3* (HG)	xYAP Δ TBS	n = 68, 83% reduced
*pax3* (HG)	xYAP Δ WW	n = 67, 54% reduced
*pax3* (HG)	xYAP Δ N-term	n = 8, 100% reduced
*pax3* (HG)	xYAP Δ C-term	n = 13, 54% reduced
*p27^xic1^*	xYAP	n = 25, 98% reduced
*p27^xic1^*	xYAP Δ TBS	n = 35, 83% reduced
*p27^xic1^*	xYAP Δ WW	n = 42, 79% reduced
*p27^xic1^*	xYAP Δ N-term	n = 35, 69% reduced
*p27^xic1^*	xYAP Δ C-term	n = 35, 69% reduced
*sox11*	xYAP	n = 35, 89% reduced
*sox11*	xYAP Δ TBS	n = 31, 90% reduced
*sox11*	xYAP Δ WW	n = 33, 82% reduced
*sox11*	xYAP Δ N-term	n = 34, 94% reduced
*sox11*	xYAP Δ C-term	n = 35, 91% reduced
*myoD*	xYAP	n = 52, 68% reduced
*myoD*	xYAP Δ TBS	n = 34, 100% reduced
*myoD*	xYAP Δ WW	n = 81, 80% reduced
*myoD*	xYAP Δ N-term	n = 71, 58% reduced
*myoD*	xYAP Δ C-term	n = 50, 19% reduced
*cyto-keratin*	xYAP	n = 32, 100% reduced
*cyto-keratin*	xYAP Δ TBS	n = 42, 100% reduced
*cyto-keratin*	xYAP Δ WW	n = 47, 100% reduced
*cyto-keratin*	xYAP Δ N-term	n = 41, 100% reduced
*cyto-keratin*	xYAP Δ C-term	n = 38, 0.03% reduced

Sample sizes and frequencies of genes that were expanded (sox2, pax3+ neural crest progenitors [NCP]) or reduced (pax3+ hatching gland [HG] progenitors, p27xic1, sox11, myoD, and cyto-keratin) after injection of wild type xyap or mutant (defined in Fig. 8A) xyap mRNAs.

## Discussion

### YAP is well conserved

Through evolution, proteins within the WW domain-containing family have functionally diversified. Although no YAP homologue exists in yeast, its closest YAP relative, Rsp5, is a WW-containing protein exhibiting ubiquitin ligase activity. The *Drosophila* YAP homologue, Yorkie, exhibits little sequence conservation when aligned with its vertebrate YAP counterparts, especially at its C-terminal end where this protein lacks the conserved vertebrate transcriptional activation domain and the SH3- and PDZ-binding motifs. However, other invertebrates, such as the acorn worm, honeybee, wasp, sea anemone, sea urchin, and sea squirt, which also exhibit low vertebrate YAP identity (∼40%), do possess the PDZ-binding motif. In order to utilize frog and fish to elucidate a common functional role in vertebrate development, it is important to establish that the YAP proteins in these animals contain similar functional domains. Indeed, xYAP and zYAP are 78% identical to the mouse homologue and contain all of the functional domains described in mammals (see also [36],[40]). Interestingly, the proline-rich region present at the N-terminus of the human homologue, which allows for hnRNP U binding, contains fewer prolines in non-mammals (human, 18; mouse, 15; frog, 6; zebrafish, 3).

The functional diversity of YAP *in vivo*, however, is just now beginning to be unraveled. In particular, there is a paucity of information regarding its function in early vertebrate developmental processes. Previously, we reported that mice lacking YAP exhibit severe developmental phenotypes that result in early lethality [15]. Given that the A-P axis defects may result from the extra-embryonic tissue defects, we exploited two more amenable models, *Xenopus laevis* and *Danio rerio*, to investigate the function of YAP during early development. Herein, we provide the first description of the mechanism by which YAP regulates the completion of gastrulation and the elongation of the A-P body axis. This protein is required for the proper timing of expression of early mesodermal genes, and for the expansion of the *sox2^+^* neural plate and *pax3^+^* neural crest progenitors at the neural plate border. We demonstrate that the effects of YAP, a transcriptional co-activator, on *pax3^+^* neural crest progenitors are accomplished, at least in part, by co-regulation of the *pax3* gene via interaction with the transcription factor, TEAD1.

### YAP is required for progression through gastrulation

We show that MO-mediated elimination of YAP *in vivo* resulted in a failure of frog and fish embryos to complete the epiboly movements of gastrulation, and a lower MO dose knockdown reduced the elongation of the A-P body axis (this study, and [36]). While germ layer inductions occurred in the absence of endogenous, zygotic xYAP protein expression, the onset of mesodermal gene expression was perturbed, indicating that YAP is required during the early steps of body axis formation. While the mechanism is not known, these results demonstrate that the similar defects described in mice lacking YAP are not simply due to nutritional deficiencies.

### Increasing YAP expands progenitors and inhibits their differentiation

When wild type *xyap*, *myap*, or *hyap* RNAs were injected into *Xenopus laevis* embryos, major morphological defects became apparent at tail bud stages. Because the tissue perturbations were widespread, we predicted that gene expression changes occurred during earlier patterning events. In fact, we observed that at neural plate stages two progenitor populations were expanded (*sox2^+^* neural plate; *pax3^+^* neural crest), whereas differentiation markers of these tissues as well as of somitic muscle and epidermis were repressed. These results are consistent with reports that over-expression of YAP expands mouse small intestinal progenitors [44] and chick neural tube *sox2* ^+^ neural progenitors [41]. The mechanism by which the expansion of neural progenitors in frog embryos is accomplished is not yet known. The expansion of mouse intestinal progenitors is mediated by activation of the Notch pathway by YAP [44]; however, frog embryos injected with *xyap* mRNA showed reduced *notch* and *hes1* RNA expression. Interestingly, xYAP did not expand all progenitors or all *pax3*-expressing cells. YAP gain-of-function inhibited *pax3* expression in hatching gland precursors, and reduced the expression of *six1*, a transcription factor that maintains the PPE in a progenitor state [52], [59]. These results demonstrate that YAP-mediated expansion of progenitor populations has tissue specificity, even within the embryonic ectoderm. The mechanisms by which YAP gain-of-function and loss-of-function both result in a shortened axis are not known. One possibility is that YAP loss-of-function leads to a loss of the progenitor pool, as we show for *sox2* and *pax3*, whereas YAP gain-of-function extends the time cells spend in a progenitor, undifferentiated state. In both cases, there would be a reduction in differentiated cells.

### xYAP directly regulates pax3 transcription

The effects of altering YAP levels on *pax3* expression in the neural crest progenitors suggested that YAP directly regulates *pax3* transcription. Increasing evidence suggests that the interaction of YAP with the TEAD family of transcription factors is critically important for proper vertebrate development, [34], [37], [41], [60], including neural progenitor expansion [41]. Therefore, we searched for highly conserved TEAD-binding sites in the 5′ regulatory region of *pax3* and found a previously undescribed, TEAD-binding site within this region. Increased expression of TEAD1 phenocopied the xYAP-mediated expansion of *pax3* in the neural crest progenitors and significantly enhanced this phenotype following coexpression of *xtead1* with levels of *xyap* mRNA that were ineffective on their own. Importantly, we demonstrated the *in vivo* relevance of this predicted association by ChIP analysis. Endogenous YAP localized to this newly identified TEAD-binding site within the 5′ regulatory region of *pax3*, but not to a region of the *pax3* promoter lacking putative TEAD-binding sites (Figure S4A). In addition, a region of the *sox2* promoter containing a putative TEAD-binding site also did not co-immunoprecipitate with YAP (Figure S4B), even though in chick neural tube YAP and TEAD1 appear to co-regulate *sox2* expression [41]. We have yet to confirm whether endogenous TEAD1 resides on this region or whether other TEADs are present. For example, there is evidence that TEAD1 and TEAD2 can functionally compensate for each other in early mouse development [61], [62]. Nonetheless, these experiments demonstrate a new developmental role for both TEAD and YAP in cooperatively driving *pax3* expression in neural crest progenitors.

Our structure/function analyses, however, indicate that the expansion of the *pax3* neural crest progenitors likely involves YAP binding to proteins in addition to TEADs, because deletion of other domains also reduced this effect. In fact, the different effects of YAP on different ectodermal and mesodermal genes appear to require different protein interaction domains, confirming that the ability of YAP to bind to multiple, diverse proteins endows this protein with multiple diverse functions. Here, we have illuminated a few key developmental roles for YAP, which appear to be consistent across three vertebrates. Moving forward, it will be interesting to see whether it is YAP's transcriptional activation abilities or its function as a scaffolding protein that is more important for each specific effect. An intriguing notion is that YAP may act as a critical scaffolding protein within the nucleus to assist in the regulation of transcription or regulate the state and/or remodeling of chromatin.

## Supporting Information

Figure S1 **mRNA and protein expression of xYAP during Xenopus laevis development.** RT-PCR analyses showed that xyap RNA was maternally expressed in an unfertilized egg and early cleavage (stage 3), decreases slightly between late cleavage (stage 6) and the mid-blastula transition (stage 9), but was then expressed abundantly through subsequent stages of Xenopus laevis development through feeding tadpole (stage 40). The (+) indicates lanes that included reverse transcriptase in the RT-PCR reaction, while the (−) indicates lanes that lacked the reverse transcriptase in the RT-PCR reaction. Western blot analysis showed that xYAP protein was maternally present at cleavage stages (stages 2–7), was detectable at the onset of epiboly and gastrulation (stages 9–10), and increased dramatically from mid-gastrula (stage 11) onwards. The (+) represents the positive control lane, which contains a cold in vitro translated xYAP product.
(TIF)

Figure S2 **Efficacy of xYAP splice blocking MOs.** xYAP splice blocking MOs (40 or 80 ng) did not completely knockdown endogenous YAP protein. YAP protein was reduced 60–66% when compared to the control MO lanes. This correlates with the xYAP splice blocking MOs causing a less penetrant open-blastopore phenotype compared to the MOs targeted to the translational start site.
(TIF)

Figure S3 **Western blot analysis confirms overexpression and proper translation of various yap mRNAs.** Using antibodies against the HA tag (left side) or hYAP (right side), immunoblots (IB) of stage 15 whole Xenopus laevis embryo lysates illustrated proper over-expression of xYAP, mYAP, and hYAP after mRNA injections at the 1-cell stage. Injected mRNAs are translated more efficiently than endogenous mRNA, accounting for an apparent lack of product in the “uninjected” lane of the YAP IB. However, see Figures 1B and S1 for endogenous YAP expression detected with this antibody.
(TIF)

Figure S4 **YAP does not co-immunoprecipitate with two other regions of Xenopus laevis genomic DNA.** (A) Another region of the pax3 promoter, not containing putative TEAD-binding sequences, failed to co-immunoprecipitate with YAP or the control IgG, yet a band of the expected size was amplified in the input lane. (B) A region of the sox2 promoter, containing a putative TEAD-binding site, did not co-immunoprecipitate with YAP or the control IgG, yet a band of the expected size was amplified in the input lane.
(TIF)
