# Supplementary figures and images for: Yes-Associated Protein 65 (YAP) Expands Neural Progenitors and Regulates Pax3 Expression in the Neural Plate Border Zone
Source: PLoS One. 2011 Jun 8;6(6):e20309. doi: 10.1371/journal.pone.0020309 (PMC3110623; doi:10.1371/journal.pone.0020309)

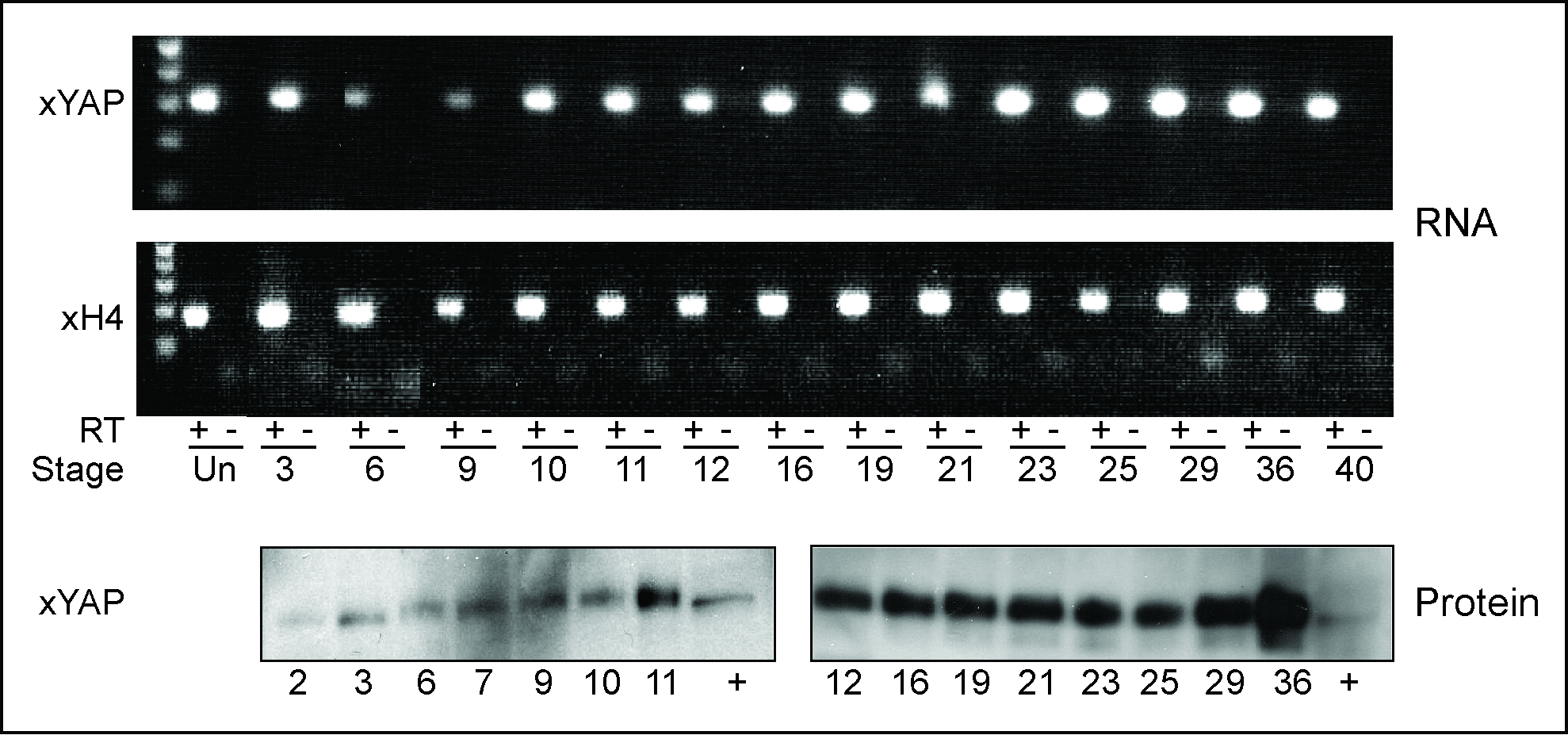

Supplement: Figure S1 — mRNA and protein expression of xYAP during Xenopus laevis development. RT-PCR analyses showed that xyap RNA was maternally expressed in an unfertilized egg and early cleavage (stage 3), decreases slightly between late cleavage (stage 6) and the mid-blastula transition (stage 9), but was then expressed abundantly through subsequent stages of Xenopus laevis development through feeding tadpole (stage 40). The (+) indicates lanes that included reverse transcriptase in the RT-PCR reaction, while the (−) indicates lanes that lacked the reverse transcriptase in the RT-PCR reaction. Western blot analysis showed that xYAP protein was maternally present at cleavage stages (stages 2–7), was detectable at the onset of epiboly and gastrulation (stages 9–10), and increased dramatically from mid-gastrula (stage 11) onwards. The (+) represents the positive control lane, which contains a cold in vitro translated xYAP product. (TIF) [file pone.0020309.s001.tif]

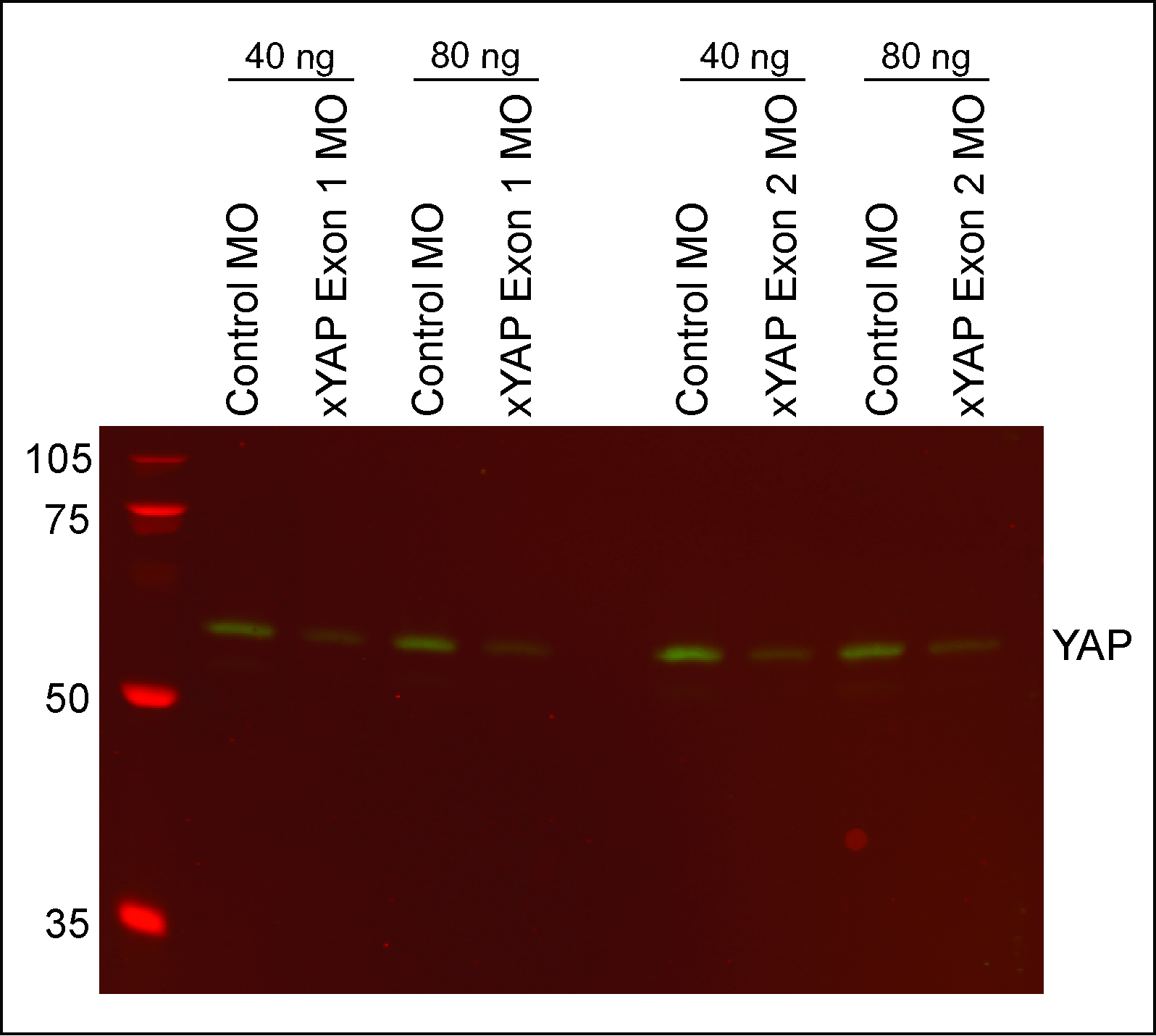

Supplement: Figure S2 — Efficacy of xYAP splice blocking MOs. xYAP splice blocking MOs (40 or 80 ng) did not completely knockdown endogenous YAP protein. YAP protein was reduced 60–66% when compared to the control MO lanes. This correlates with the xYAP splice blocking MOs causing a less penetrant open-blastopore phenotype compared to the MOs targeted to the translational start site. (TIF) [file pone.0020309.s002.tif]

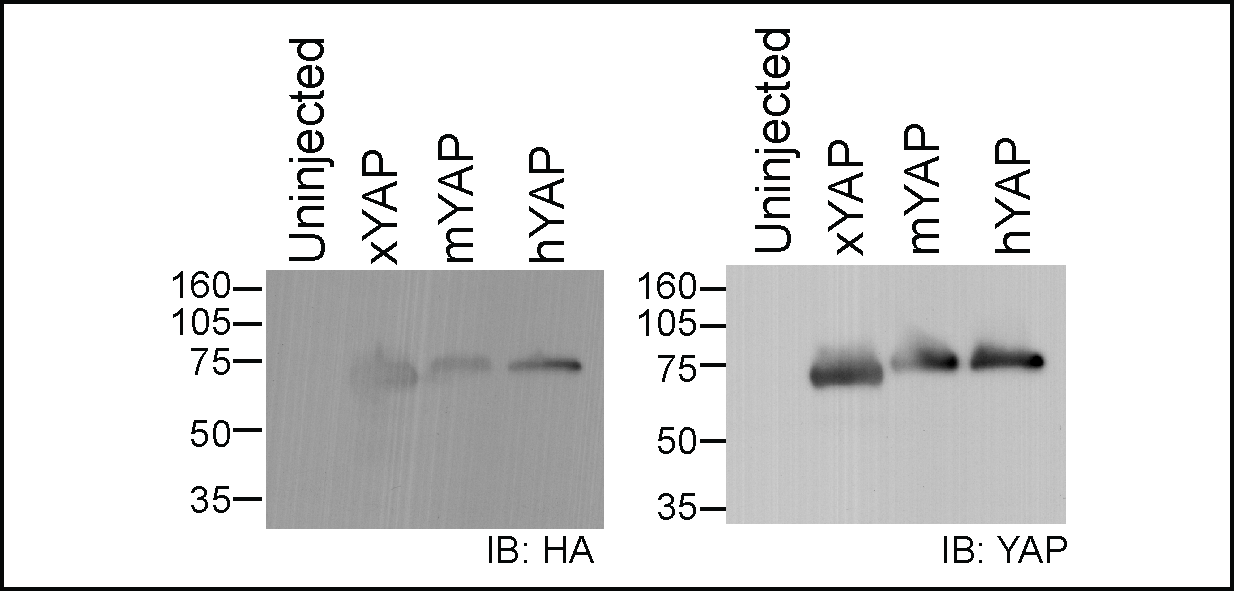

Supplement: Figure S3 — Western blot analysis confirms overexpression and proper translation of various yap mRNAs. Using antibodies against the HA tag (left side) or hYAP (right side), immunoblots (IB) of stage 15 whole Xenopus laevis embryo lysates illustrated proper over-expression of xYAP, mYAP, and hYAP after mRNA injections at the 1-cell stage. Injected mRNAs are translated more efficiently than endogenous mRNA, accounting for an apparent lack of product in the “uninjected” lane of the YAP IB. However, see Figures 1B and S1 for endogenous YAP expression detected with this antibody. (TIF) [file pone.0020309.s003.tif]

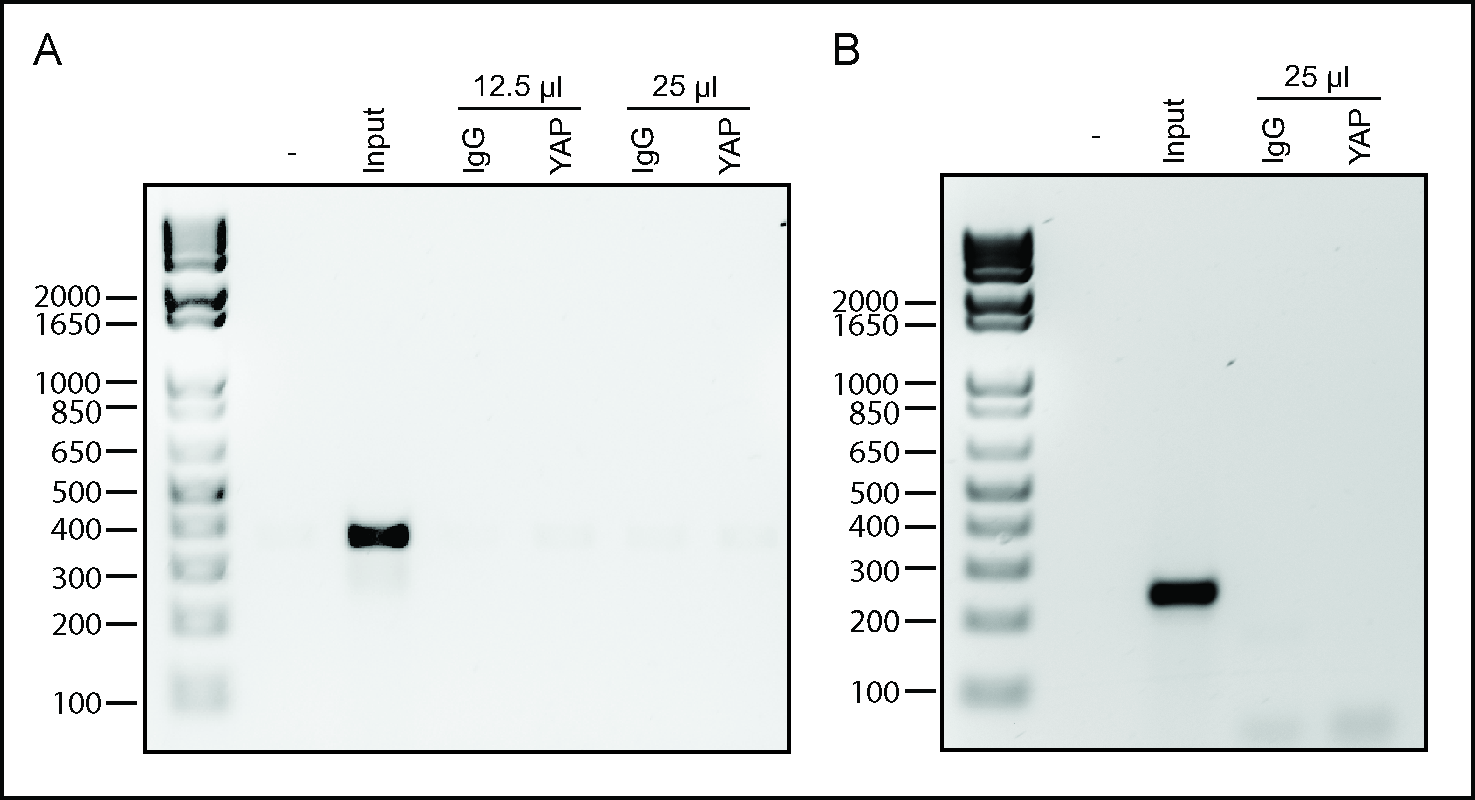

Supplement: Figure S4 — YAP does not co-immunoprecipitate with two other regions of Xenopus laevis genomic DNA. (A) Another region of the pax3 promoter, not containing putative TEAD-binding sequences, failed to co-immunoprecipitate with YAP or the control IgG, yet a band of the expected size was amplified in the input lane. (B) A region of the sox2 promoter, containing a putative TEAD-binding site, did not co-immunoprecipitate with YAP or the control IgG, yet a band of the expected size was amplified in the input lane. (TIF) [file pone.0020309.s004.tif]
